# Merosin-deficient congenital muscular dystrophy type 1a: detection of *LAMA2* variants in Vietnamese patients

**DOI:** 10.3389/fgene.2023.1183663

**Published:** 2023-06-14

**Authors:** Van Khanh Tran, Ngoc-Lan Nguyen, Lan Ngoc Thi Tran, Phuong Thi Le, Anh Hai Tran, Tuan L. A. Pham, Nguyen Thi Kim Lien, Nguyen Thi Xuan, Le Tat Thanh, Thanh Van Ta, Thinh Huy Tran, Huy-Hoang Nguyen

**Affiliations:** ^1^ Center for Gene and Protein Research, Hanoi Medical University, Hanoi, Vietnam; ^2^ Institute of Genome Research, Vietnam Academy of Science and Technology (VAST), Hanoi, Vietnam; ^3^ Hanoi Medical University Hospital, Hanoi Medical University, Hanoi, Vietnam; ^4^Graduate University of Science and Technology, Vietnam Academy of Science and Technology (VAST), Hanoi, Vietnam

**Keywords:** merosin-deficient congenital muscular dystrophy type 1A, MDC1A, LAMA2 gene, Vietnamese, congenital muscular dystrophy

## Abstract

**Background:** Merosin-deficient congenital muscular dystrophy type 1A (MDC1A), also known as laminin-α2 chain-deficient congenital muscular dystrophy (*LAMA2*-MD), is an autosomal recessive disease caused by biallelic variants in the *LAMA2* gene. In MDC1A, laminin- α2 chain expression is absent or significantly reduced, leading to some early-onset clinical symptoms including severe hypotonia, muscle weakness, skeletal deformity, non-ambulation, and respiratory insufficiency.

**Methods:** Six patients from five unrelated Vietnamese families presenting with congenital muscular dystrophy were investigated. Targeted sequencing was performed in the five probands. Sanger sequencing was carried out in their families. Multiplex ligation-dependent probe amplification was performed in one family to examine an exon deletion.

**Results:** Seven variants of the *LAMA2* (NM_000426) gene were identified and classified as pathogenic/likely pathogenic variants using American College of Medical Genetics and Genomics criteria. Two of these variants were not reported in the literature, including c.7156-5_7157delinsT and c.8974_8975insTGAT. Sanger sequencing indicated their parents as carriers. The mothers of family 4 and family 5 were pregnant and a prenatal testing was performed. The results showed that the fetus of the family 4 only carries c.4717 + 5G>A in the heterozygous form, while the fetus of the family 5 carries compound heterozygous variants, including a deletion of exon 3 and c.4644C>A.

**Conclusion:** Our findings not only identified the underlying genetic etiology for the patients, but also provided genetic counseling for the parents whenever they have an offspring.

## Introduction

Congenital muscular dystrophies (CMDs) are phenotypically heterogeneous diseases and characterized by early muscle weakness and hypotonia presenting early after birth or infancy (Pasrija and Tadi, 2022). CMDs are rare, with an incidence of 0.82/100,000 live births (Pasrija and Tadi, 2022). The most common types of CMDs involve collagen type VI related, laminin-α2 related, and alpha dystroglycan related groups. Recently, Zambo and Muntoni showed that thirty-seven genes have been reported to be associated with CMDs (Zambon and Muntoni, 2021).

Laminin-α2 chain-deficient muscular dystrophy is the most common form of congenital muscular dystrophy worldwide, which affects about 30% of CMD patients in Europe (Mostacciuolo et al., 1996; Allamand and Guicheney, 2002; Geranmayeh et al., 2010), 48% in Qatar (Abdel Aleem et al., 2020), 36.4% in China (Ge et al., 2019), and 16% in Australia (O’Grady et al., 2016). The disease is an autosomal recessive disorder with the reduction or absence in the production of protein laminin-α2 which is caused by *LAMA2* gene mutations (OMIM *156225) (Helbling-Leclerc et al., 1995). The deficiency or absence of the laminin-α2 subunit leads to a lack of laminin-211 and/laminin-221 and results in a reduced strength and stability of skeletal muscle tissue. Depending on the extent of laminin-α2 deficiency, the clinical manifestations of *LAMA2* related muscular dystrophy (*LAMA2*-MD) range from severe congenital muscular dystrophy type 1A (MDC1A, OMIM#607855) to milder late-onset *LAMA2*-MD (OMIM#618138). Newborns with severe MDC1A are characterized by a weak cry, muscle weakness and hypotonia, leading to delayed motor developmental milestones (Jones et al., 2001; Gilhuis et al., 2002). Most MDC1A children lose independent ambulation, only a few could walk with assistance (Durbeej, 2015). The growth development is also restricted due to patients suffering from feeding difficulties, chewing, and swallowing issues (Philpot et al., 1999). Enteral feeding was required for several MDC1A patients (Geranmayeh et al., 2010). MDC1A patients may develop respiratory failure and occasionally require ventilatory assistance in their life (Geranmayeh et al., 2010). Respiratory tract infection is the leading cause of death in 30% of children with early-onset MDC1A in the first decade of life (Wallgren-Pettersson et al., 2004). Affected individuals may develop facial muscle weakness and macroglossia, resulting in myopathic facies with an open mouth and protruded tongue (Oliveira et al., 2020). Elevated serum creatine kinase (CK) levels, immunohistochemistry of skin or muscle biopsies, and white matter abnormalities on brain MRI could be used for diagnosis of MDC1A (Previtali and Zambon, 2020; Sarkozy et al., 2020). However, immunohistochemistry is not available in some laboratories (Tran et al., 2020; El Kadiri et al., 2021). In several cases, immunohistochemistry and brain MRI might not be sufficient for an accurate diagnosis of *LAMA2*-MD. Genetic testing using next-generation sequencing is recommended for *LAMA2*-MD (Saredi et al., 2019; El Kadiri et al., 2021).

**FIGURE 1:**
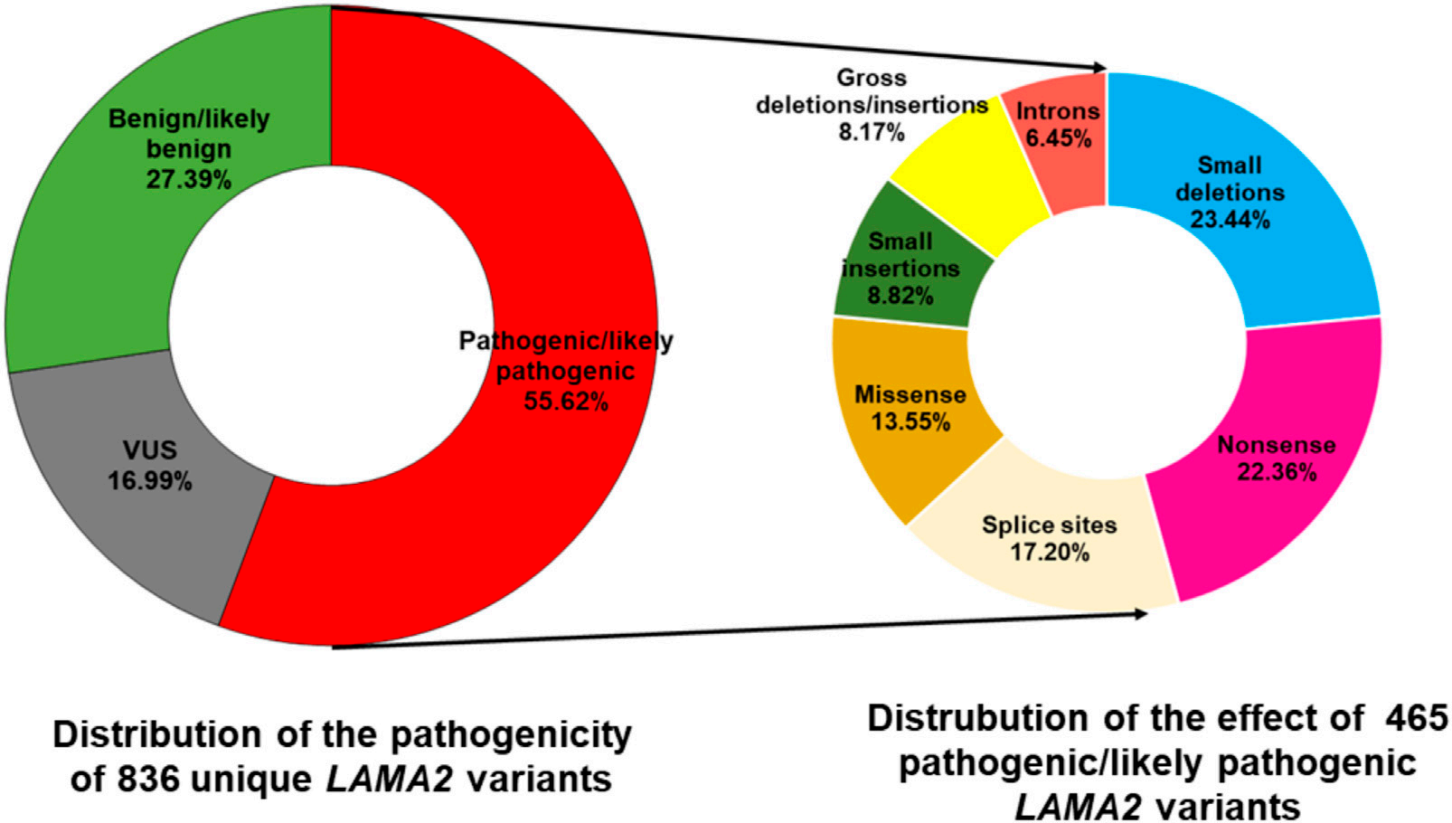
Distribution of pathogenicity of 836 unique *LAMA2* variants and the effect of 465 pathogenic/likely pathogenic variants. The data was accessed in the LOVD database on 19 February 2023. VUS, variant of uncertain significance. Splice sites variants are alterations occurring at the start two nucleotides or the last two nucleotides of an intron.

The *LAMA2* gene is located on chromosome 6q22.33 and consists of 65 exons (Allamand and Guicheney, 2002). There are 836 unique variants that have been reported in the *LAMA2* in the LOVD database (https://databases.lovd.nl/shared/variants/LAMA2/unique; accessed on 18 February 2023), of which, 465 (55.62%) were classified as pathogenic/likely pathogenic variants, 142 (16.99%) were variant of uncertain significance, and 229 (27.39%) were benign/likely benign variants (Supplementary Table S1; Figure 1). The pathogenic/likely pathogenic variants involve 109 (23.44%) deletions, 104 (22.36%) nonsense, 79 (16.99%) splice sites, 63 (13.55%) missense, 41 (8.82%) insertions, 39 (8.39%) gross deletions/insertions, and 30 (6.45%) introns (Supplementary Table S1 and Figure 1). Truncated protein variants contributed half of pathogenic/likely pathogenic variants (246/465, 52.90%). Exon 4 contains most variants (30 variants), however, only one was classified as a benign variant (Supplementary Table S1). Therefore, exon 4 may be a hotspot area for pathogenic/likely pathogenic variants. Most of the variants (444/465, 95.48%) have been published in the literature. Numerous *LAMA2* variants are recurrent (Supplementary Table S1). The most common variants which have been reported over 20 times include c.2049_2050delAG (54 reported), c.3942 + 2T>C (34 reported), c.3976C>T (31 reported), and c.3085C>T (25 reported) (Supplementary Table S1) (Pegoraro et al., 1998; Geranmayeh et al., 2010; Oliveira et al., 2018; Ge et al., 2019).

Four pathogenic/likely pathogenic *LAMA2* variants have been reported in Vietnamese patients with muscular dystrophy, including c.778C>T (p.H260Y) and c.2987G>A (p.C996Y) (Nguyen et al., 2020), c.1964T>C (p.L655P) and c.3556-13T>A (Tran et al., 2020). However, a large number of *LAMA2* variants can be further detected in Vietnamese patients with MDC1A. In this study, we describe six unrelated patients presenting with congenital muscular dystrophy. Targeted next-generation sequencing and multiplex ligation-dependent probe amplification (MLPA) were performed to achieve an accurate molecular diagnosis. Genetic counseling was performed for the five families. Invasive prenatal testing was performed on two families.

## Patients and methods

### Patients

This study included six patients who presented with hypotonia and muscle weakness early in life. Two of the six patients are siblings. The patients were from Hanoi Medical University Hospital. All patients were from non-consanguineous families.

### Target sequencing and data analysis

DNA samples of the patients and family members were extracted from peripheral blood using QIAmp® DNA Mini Kit. We used NanoDrop™ 2000/2000c spectrometer (Thermo, United States) to measure the purity and concentration of the results. Targeted amplicon sequencing using a gene panel for hereditary muscular dystrophies on the Illumina HiSeq 2000 platform (Illumina Inc., San Diego, CA) was performed on patients 1–5. The variants with minor allele frequency greater than 1% according to the 1000 Genomes Project database were excluded. The remaining variants interpreted as pathogenic variants by ClinVar (http://www.clinvar.com/); exonic non-synonymous, coding insertions or deletions, and splice site were screened. The single heterozygous variants in the recessive inheritance genes were removed.

The pathogenicity of identified variants was predicted using MutationTaster 2021 (Steinhaus et al., 2021) and CADD v1.6 (Rentzsch et al., 2021). Splice-site prediction was performed using varSEAK (https://varseak.bio/).

### Sanger sequencing, multiplex ligation-dependent probe amplification (MLPA), and prenatal testing

Sanger sequencing was performed for the segregation analysis using the suitable primers (Supplementary Table S2) for *LAMA2* obtained from previous research (Guicheney et al., 1998). Sequencing was done using ABI PRISM 3500 Genetic Analyser (Model 373A, Applied Biosystems, CA, United States).

MLPA was analyzed to identify an exon deletion in patient 5’s family using the SALSA MLPA Probemix P391 LAMA2 mix 1 version A3 as described by Cauley et al. (2020).

Prenatal testing was offered to the families of patient 4 and patient 5 since the mothers were pregnant. The identified variants were checked in the amniotic fluid obtained at 17 weeks of pregnancy.

### Interpretation of variant pathogenicity

The pathogenicity of identified variants was interpreted according to ACMG guidelines (Richards et al., 2015). Each pathogenic criterion is weighted as very strong (PVS1), strong (PS1–4); moderate (PM1–6), or supporting (PP1–5). Each benign criterion is weighted as stand-alone (BA1), strong (BS1–4), or supporting (BP1–6). Based on the five-tier classification system, variants are classified as pathogenic, likely pathogenic, benign, likely benign, and uncertain significance variants.

## Results

**FIGURE 2:**
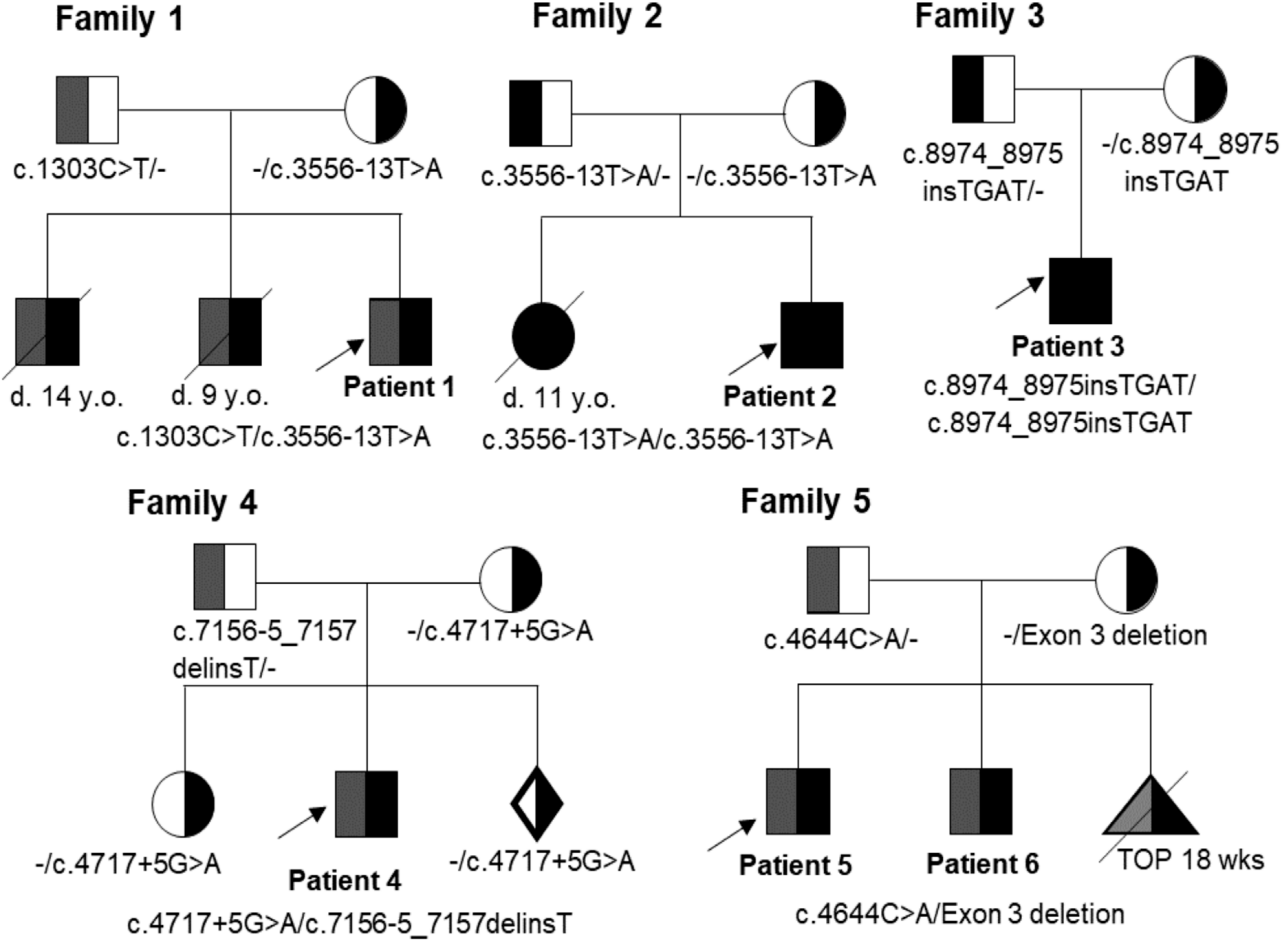
Pedigrees of families 1-5 with variants in the *LAMA2* gene. Probands are indicated by arrows. Affected individuals are shown in filled symbols. D: Deceased; y. o.: Year-old; TOP: Termination Of Pregnancy.

Six patients with CMD in Vietnam were identified (Table 1). The onset of symptoms and musculature involvements of all cases occurred before six-months-old. None of them showed any respiratory distress or dysfunction of the autonomic nervous system. In the last visit, when the age was > 2-years-old, the six patients showed inability to walk or wheelchair-dependence. The families 1, 2, and 5 showed family history of CMD (Table 1; Figure 2). Patient 1 presented with weak cry and muscular hypotonia at birth, and slight hypotonia in both legs at 3 months of age. His brothers presented with the same symptoms and died at the age of 14 years and 9 years. Patient 2 showed a proximal muscle weakness in lower limbs and weak movements from 5 months of age. His elder sister had similar phenotypes with him and died at 11 years of age. Patient 3 had slight hypotonia in both legs at 3 months of age and proximal muscle weakness in upper and lower limbs at 5 months of age. Patient 3 is the first child of the family. Patient 4 presented with weak cry at birth and neck flexor weakness at 4 months of age. He had typical elongated myopathic face, wide mouth, and protruded tongue. His elder sister did not have any muscular dystrophy symptoms. Patient 5 and patient 6 are siblings. Patients 5 and 6 showed hypotonia from one-month-old and 12-day-old, respectively. Patients 5 and 6 were able to sit from 8-month-old but could not walk without support. Both patients presented with a typical elongated myopathic face. The magnetic resonance imaging of patient 6 showed white matter hyperintensities lesions on T2WI and T2 Flair.

**TABLE 1:** Clinical characteristics and LAMA2 variants identified in the six patients.

	Patient 1	Patient 2	Patient 3	Patient 4	Patient 5	Patient 6
Clinical characteristics						
First symptoms	From birth: weak cry, muscular hypotonia	3 months: muscle weakness, weak movements	4 months: slight hypotonia in both legs	From birth: weak cry	1 months: hypotonia	12 days: hypotonia
Family history	Two brothers died at 14 years and 9 years with the same symptoms	One sister died at 11 years with the same symptoms	-	One sister with normal phenotype	One alive brother (Patient 6) with similar muscle weakness symptoms	One alive brother (Patient 5) with similar muscle weakness symptoms
Apgar score	7 (weak cry)	10	10	7 (weak cry)	10	10
Respiratory distress	No	No	No	No	No	No
Musculature	3 months: slight hypotonia in both legs	5 months:	5 months:	4 months: Neck flexor weakness	6 months: hard to roll over 8 months: able to sit	8 months: able to sit
		Proximal muscle weakness in lower limbs	Proximal muscle weakness in upper and lower limbs			
Facial Dysmorphy	No	No	No	Typical elongated myopathic face, with an open mouth and tongue protrusion	Typical elongated myopathic face	Typical elongated myopathic face
Dysfunction of ANS	No	No	No	No	No	No
Creatine kinase level	Elevated	Elevated	Elevated	Elevated	Elevated	Elevated
MRI	NA	NA	NA	NA	NA	White matter hyperintensities lesions on T2WI and T2 Flair
Last clinical follow-up.	Alive, 2 years, unable to walk	Alive, 3 years, unable to walk	Alive, 5 years, able to sit, wheelchair dependence	Alive, 4 years, able to sit with his hands as support, wheelchair dependence	Alive 10 years, able to sit, wheelchair dependence	Alive 8 years, able to sit, wheelchair dependence
*LAMA2* variants (NM_000426.3)						
Patient	c.1303C>T, het	c.3556–13T>A, hom	c.8974_8975insTGAT, hom	c.4717 + 5G>A, het	Exon 3 del, het c.4644C>A, het	Exon 3 del, het c.4644C>A, het
	c.3556–13T>A, het			c.7156–5_7157delinsT, het		
Paternal	c.1303C>T, het	c.3556–13T>A, het	c.8974_8975insTGAT, het	c.7156–5_7157	c.4644C>A, het	c.4644C>A, het
				delinsT, het		
Maternal	c.3556–13T>A, het	c.3556–13T>A, het	c.8974_8975insTGAT, het	c.4717 + 5G>A, het	Exon 3 del, het	Exon 3 del, het

Abbreviation: het: heterozygous; hom: homozygous; ANS: autonomic nervous system, MRI: magnetic resonance imaging; NA: not analyzed.

Targeted sequencing identified seven *LAMA2* variants in the five probands (Patients 1–5, Table 1). Two of these variants were not previously reported in the literature, including *LAMA2* (NM_000426): c.7156-5_7157delinsT and c.8974_8975insTGAT (Table 2). These variants were predicted as deleterious variants in MutationTaster21 and classified as pathogenic variants according to ACMG.

**TABLE 2:** Evaluation of the seven LAMA2 (NM_000426) variants.

c.DNA change	Phys. Location (exon)	AA change	Frequency	dbSNP	ClinVar ID	HGMD	Mutation Taster	CADD	ACMG	Literature
Deletion Exon 3	Exon 3	−	−	−	−	CG1417642	−	−	Pathogenic (PVS1, PS3, PM2, PM4, PP1, PP4)	Reported
c.1303C>T	chr6:129486817C>T (Exon 9)	p.R435*	2/264690–1/3,854, het	rs773209126	235805 Pathogenic	Pathogenic CM102052	Del	37	Pathogenic (PVS1, PS1, PM2, PM3, PM4, PP3, PP4)	Reported
c.3556–13T>A	chr6:129636608T>A (Intron 24–25)	p.V1186Tfs*4	1/264690–5/251408, het	rs775278003	555549 Likely pathogenic	Pathogenic CS151380	Del	22.5	Likely pathogenic (PM2, PM3, PP3, PP4, PP5)	Reported
c.4644C>A	chr6:129674429C>A (Exon 32)	p.C1548*	−	−	−	−	Del	37	Pathogenic (PVS1, PM2, PM3, PM4, PP3, PP4)	Reported
c.4717 + 5G>A	chr6:129674507G>A (Intron 32–33)	−	1/251308–3/264690, het	rs776050258	1028253 VUS	−	Del	25	Likely pathogenic (PM2, PM3, PP3, PP4)	Reported
c.7156-5_7157delinsT	chr6:129786285_129786291delinsT (Intron 50–51+Exon 51)	R2386*	−	rs1554301854	477503 Pathogenic	−	Del	−	Pathogenic (PVS1, PM2, PM3, PM4, PP3, PP4)	Not
c.8974_8975insTGAT	chr6:129833624_129833625insTGAT (exon 63)	p.E2995*	−	−	−	−	Del	−	Pathogenic (PVS1, PM2, PM3, PM4, PP3, PP4)	Not

Abbreviation: het–heterozygous, Del-Deleterious, VUS-Variant of Uncertain Significance, HGMD-Human Gene Mutation Database, PVS-pathogenic very strong, PS-pathogenic strong, PM-pathogenic moderate, PP-pathogenic supporting.

Patient 1 carried compound heterozygous variants including c.1303C>T (p.R435*) that was maternally inherited, and c.3556–13T>A (p.V1186Tfs*4) that was paternally inherited (Table 1; Figure 2). The variant c.1303C>T was reported as a pathogenic variant in ClinVar (ID 235805) and HGMD (CM102052) (Table 2). The variant c.3556-13T>A was reported as a likely pathogenic variant in ClinVar (ID 555549) (Table 2). Both variants are rare, with a frequency of 2/264690-1/3,854 for c.1303C>T (p.R435*) (https://www.ncbi.nlm.nih.gov/snp/rs773209126, accessed on 18 February 2023) and a frequency of 1/264690-5/251408 for c.3556-13T>A (p.V1186Tfs*4) (https://www.ncbi.nlm.nih.gov/snp/rs775278003, accessed on 18 February 2023) (Table 2). The c.1303C>T produces a premature stop codon from arginine at the amino acid position 435 (p.R435*). The patient 2 harbored the variant c.3556–13T>A in the homozygous state, while his parents carried the variant in the heterozygous state (Table 1; Figure 2).

**FIGURE 3:**
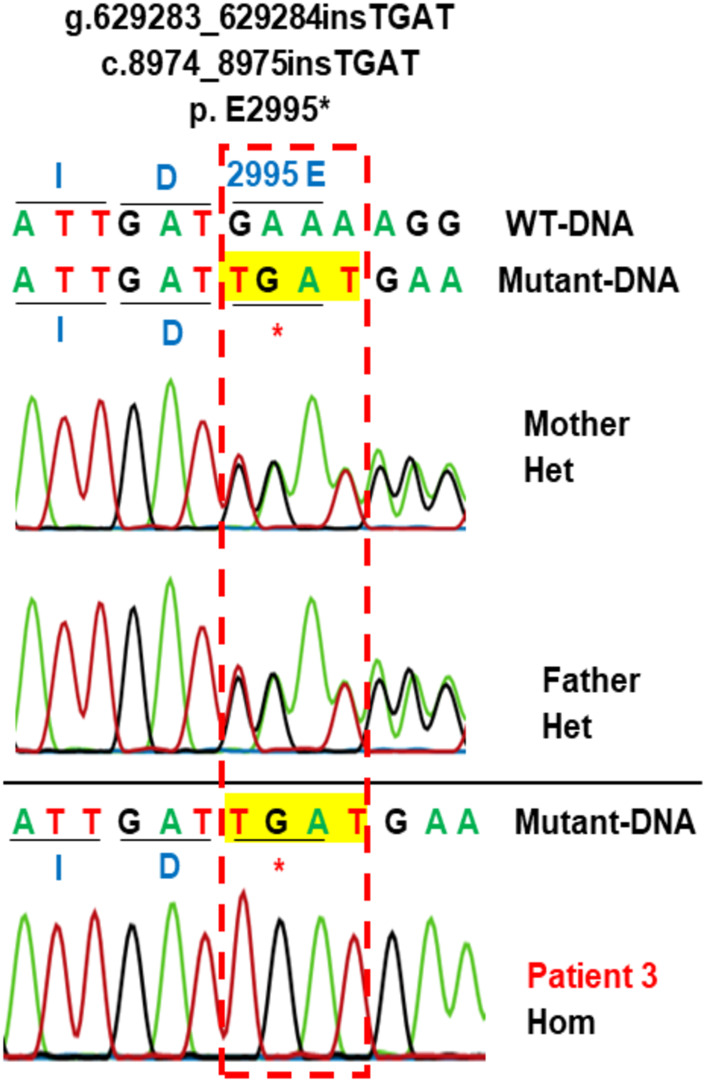
Sanger sequencing analysis of the variant c.8974_8975insTGAT in the family 3. Patient 3 carries variant c.8974_8975insTGAT (g.629283_629284insTGAT) in the homozygous state. The parents carry the variant in the heterozygous state. This variant creates a new stop codon at the position amino acid number 2995 (p.E2995*). The protein is truncated. Hom, homozygous; Het, heterozygous; WT, wild-type.

Patient 3 carried the variant c.8974_8975insTGAT in the homozygous state (Table 1; Figure 3). The parents carried the variant in the heterozygous state. The variant was predicted to cause a frameshift of the amino acid sequence of LAMA2 and a gain of stop in a truncated protein at the position glutamic acid 2,995 (p.E2995*). This variant was not reported in the literature, in the LOVD, gnomAD, ExAC, dbSNP155, ClinVar, HGMD or in-house databases. The variant was classified as a pathogenic variant according to ACMG with PVS1, PM2, PM3, PM4, PP3, and PP4 supporting (Table 2).

**FIGURE 4:**
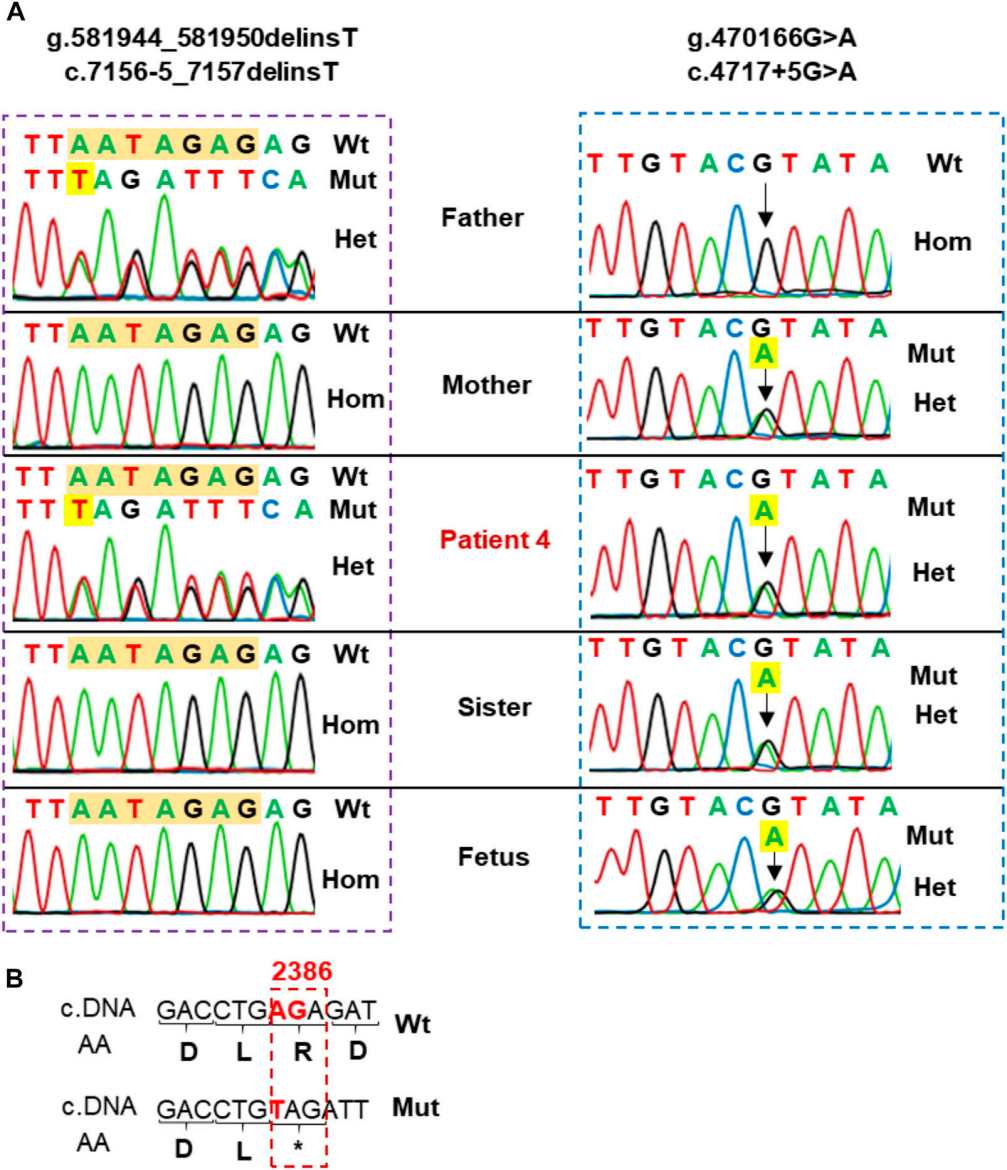
**(A)** Sanger sequencing analysis of the family 4. Patient 4 carried two variants, including c.7156–5_7157delinsT inherited from the father and c.4717 + 5G>A inherited from the mother. His sister and fetus only carried c.4717 + 5G>A in the heterozygous state. **(B)** Sequence comparison between wild type and variant c.7156-5_7157delinsT. The variant alters the sequence at the intersection of intron 50 and exon 51 and may produce a premature termination of the protein containing 2,386 amino acids (p.R2386*).

Patient 4 presented with compound heterozygous *LAMA2* variants, c.4717 + 5G>A that was maternally inherited and c.7156-5_7157delinsT that was paternally inherited (Table 1; Figure 4). The variant c.4717 + 5G>A was predicted to be a likely splicing effect by the varSEAK tool with class 4. Based on the ACMG guidelines and segregation analysis in family 4, c.4717 + 5G>A was interpreted as a likely pathogenic variant with two moderate supporting (PM2 and PM3) and two possible supporting (PP3 and PP4). The variant c.7156-5_7157delinsT deletes the last 5 nucleotides of intron 5 and the first 2 nucleotides of exon 51 and inserts a T nucleotide. The varSEAK tool predicted the variant c.7156–5_7157delinsT to have a class 5 splicing effect. This mutation is predicted to lead to premature termination of the protein that consists of 2,386 amino acids (p.R2386*). The variant c.7156-5_7157delinsT was reported as a pathogenic variant in the ClinVar (ID 477503), however, not in the literature. During our investigation, the mother of the family had a pregnancy and decided to have a prenatal diagnosis. The mother had amniocentesis at a 17-week gestational age. The fetus was found to be heterozygous for c.4717 + 5G>A and the pregnancy continued.

**FIGURE 5:**
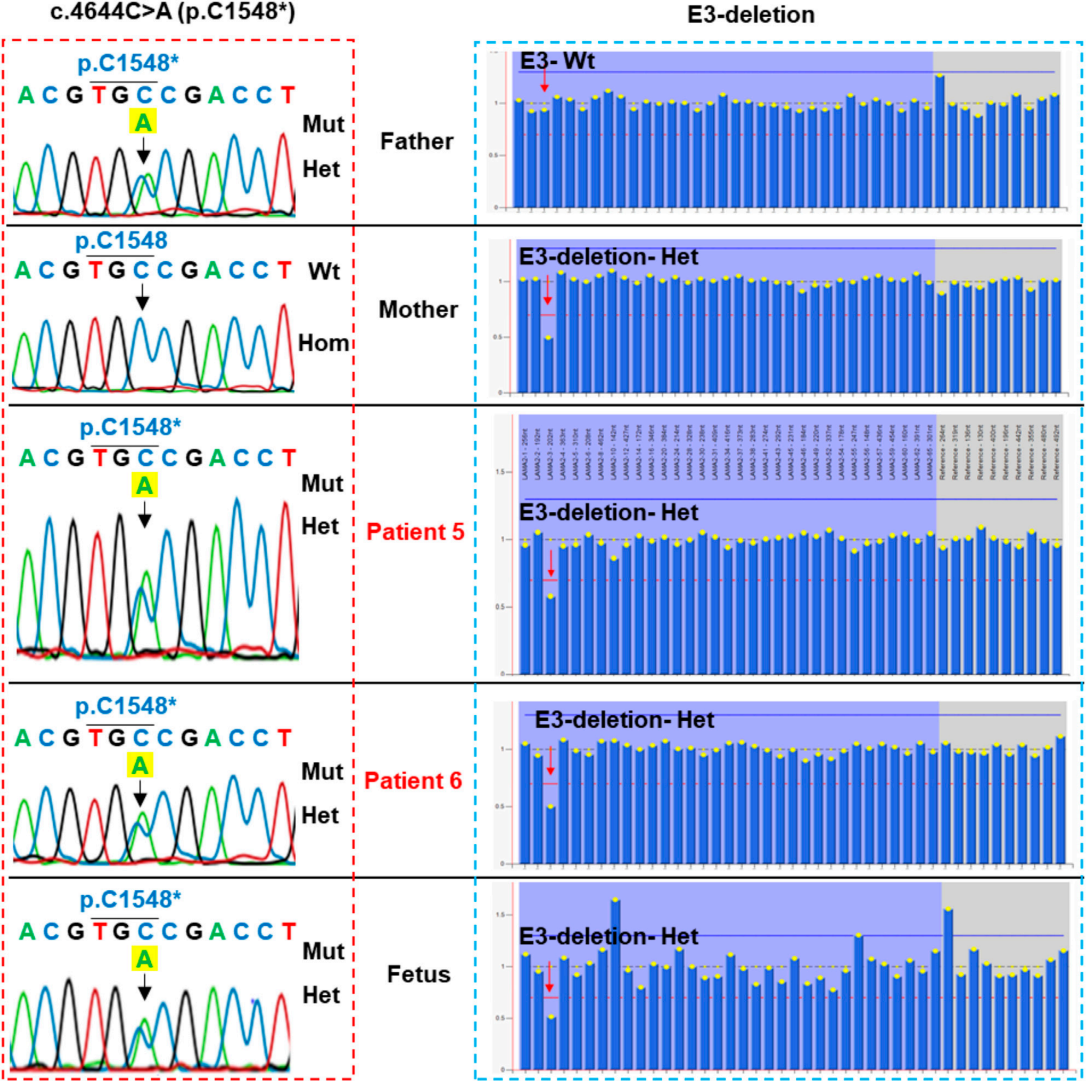
Sanger sequencing analysis and multiplex ligation-dependent probe amplification (MLPA) of the family of patient 5. Patient 5, his brother and the fetus carried compound heterozygous variants, including c.4644C>A (p.C1548*) inherited from the father (the left panel) and a deletion of exon 3 inherited from the mother (the right panel). Mut, mutation; Hom, homozygous; Het, heterozygous.

Patient 5 carried a truncating variant c.4644C>A (p.C1548*) that was paternally inherited and a deletion of exon 3 that was maternally inherited (Table 1; Figure 5). The deletion of exon 3 was interpreted as a pathogenic variant with PVS1, PS3, PM2, PM4, PP1, and PP4 supporting (Table 2). The c.4644C>A creates a premature stop codon at amino acid 1,548 of the LAMA2 protein. The variant c.4644C>A (p.C1548*) is classified as a pathogenic variant with PVS1, PM2, PM3, PM4, PP3, and PP4 supporting. The mother of patient 5 had amniocentesis at a 17-week gestational age and performed genetic testing. The result showed that the fetus harbored both c.4644C>A (p.C1548*) and deletion of exon 3, like his brother (Figure 4).

## Discussion

The clinical symptoms of six patients in this study occurred early (before 6-months-old), in agreement with suggestive findings by Oliveira et al. (Oliveira et al., 1993). They all suffered from significant hypotonia and the inability to walk from an early period of life.

**FIGURE 6:**
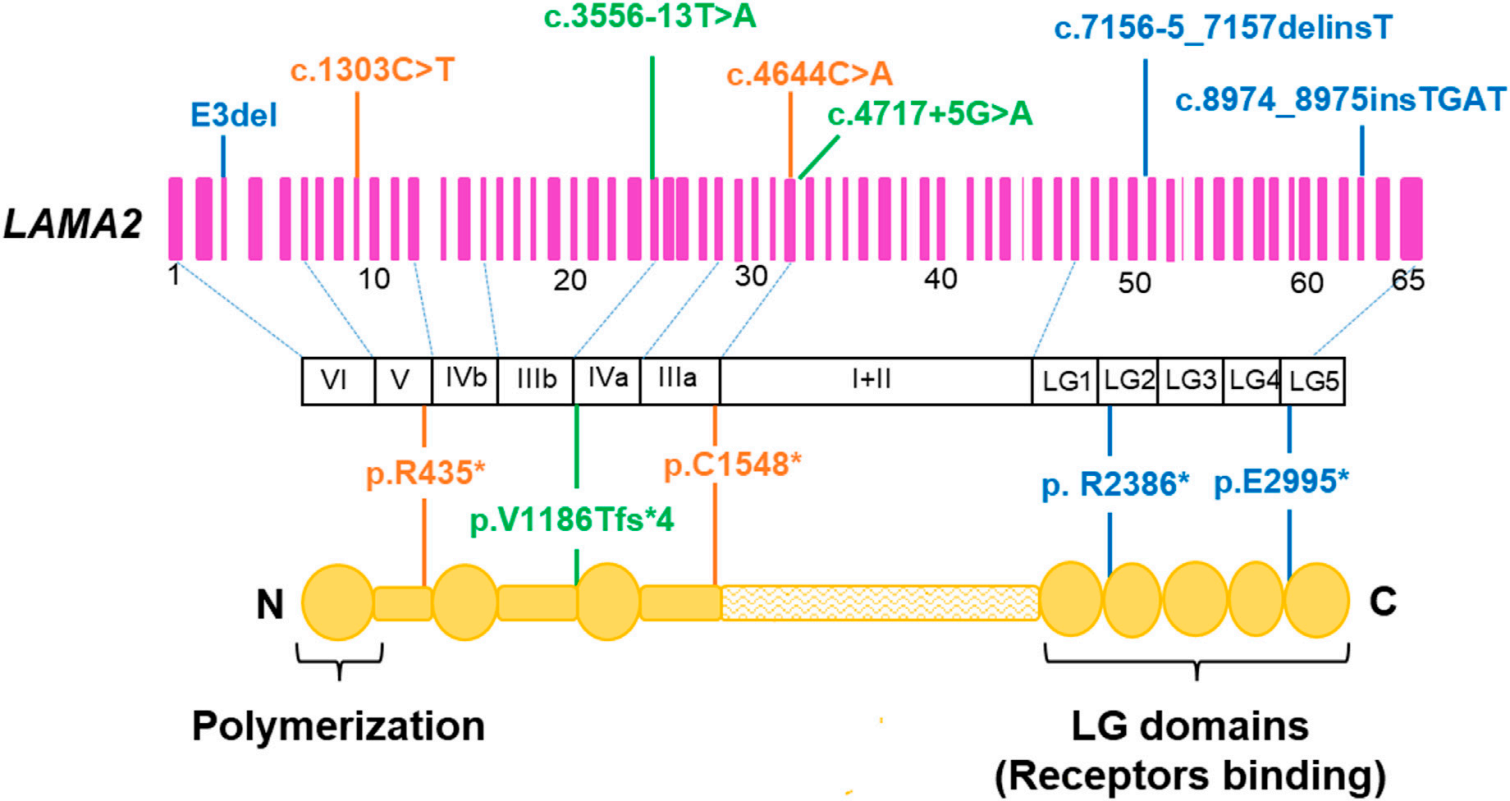
Location of the seven identified variants in the *LAMA2* gene and protein. Deletion/insertion variant are shown in blue, nonsense variants in orange, and intron variants in green. Adapted to Zambon et al. (2020).

In this study, 5/7 variants were predicted to result in a truncated protein (Table 2; Figure 6), which is in line with the previous studies (Oliveira et al., 2008; Oliveira et al., 2018; Geranmayeh et al., 2010; Xiong et al., 2015), in which, variants predicted to lead to premature termination of translation in *LAMA2* are the most common variants identified in patients with MDC1A. Laminin-α2 is a part of the laminin-211which form a link between the sarcolemma of muscle fibers and the extracellular matrix. The five LG domains of laminin-α2 play as receptor binding sites (Holmberg and Durbeej, 2013). The LG1-3 domains and LG4-5 domains are responsible for binding to intergrin α7β1 and α-dystroglycan, respectively. The extracellular α-dystroglycan is anchored to the cell transmembrane by linking to β-dystroglycan. The β-dystroglycan binds to dystrophin which is coupled to the actin-rich cytoskeleton (Campbell, 1995). Therefore, the LG4-5 domains are critical for the α-dystroglycan/laminin interaction, which plays an important role for the stability of basement membranes. Truncating variants causing the lack of the LG domains frequently result in the loss of function of LAMA2 and cause the disease. In fact, based on the LOVD database, all truncating variants have been classified as pathogenic/likely pathogenic variants (Supplementary Table S1). In our study, all patients carried at least a truncating pathogenic/likely pathogenic variant, therefore, the patients presented symptoms early in life. None of the seven identified variants were missense, while another Vietnamese patient with MDC1A harbored a missense mutation c.1964T>C (p.L655P) and c.3556-13T>A (Tran et al., 2020). Two missense variants, c.778C>T (p.H260Y) and c.2987G>A (p.C996Y), have been reported in Vietnamese patients with late-onset *LAMA2*-MD who achieved independent walking (Nguyen et al., 2020). This result is consistent with the findings of Oliveira et al. (2018) and Tan et al. (2021), where missense variants were more frequent in late-onset *LAMA2*-MD than MDC1A.

The variant c.3556-13T>A was detected in patients 1 and 2 in our study, and another reported Vietnamese patient with MDC1A (Tran et al., 2020). The variant c.3556-13T>A was predicted to cause a premature termination of transcription (p.V1186Tfs*4) (Xiong et al., 2015). Patient 2 carried c.3556-13T>A in the homozygous state, while patient 1 and the individual in the study of Tran et al. harbored c.1303C>T (p.R435*) and c.1964T>C (p.L655P), respectively. Mutation c.1303C>T (p.R435*) was published as a nonsense mutation and expected to produce a truncated protein, thus, leading to a loss of protein function (Geranmayeh et al., 2010). The variant c.3556–13T>A was reported in two Chinese siblings (Xiong et al., 2015; Ge et al., 2019; Tan et al., 2021). The two siblings carried another frameshift variant, c.4883delC (p.P1629Qfs*11) (Xiong et al., 2015). There are several similar clinical characteristics between these MDC1A cases, such as ability to sit but inability to walk, normal intelligence, and progressive scoliosis.

Patient 3 carried a novel homozygous variant c.8974_8975insTGAT (p.E2995*) which was located in exon 63 and predicted to truncate 172 amino acids located in the last domain LG5 of LAMA2 (Figure 5). Exon 63 might play an important role in the function of the G-like domain (Tan et al., 2021). The pathogenicity of this novel variant is supported by data in the literature that reported c.8987del (p.K2996Sfs*2) causing the creation of a stop codon next to p.E2995* (Tan et al., 2021).

Interestingly, in this study we reclassified a VUS (c.4717 + 5G>A) as a likely pathogenic variant based on segregation analysis. The family segregation analysis has been considered as an effort method to reclassify a VUS as pathogenic/likely pathogenic or benign/likely pathogenic (Garrett et al., 2016; Rush et al., 2022). The variant c.4717 + 5G>A was reported as uncertain significance in the ClinVar database (ID 1028253) by Baylor Genetics and Invitae submitters but not in the literature. Our study is the first report of c.4717 + 5G>A as a likely pathogenic variant in a MDC1A patient. The variant c.4717 + 5G>A affects a nucleotide within the consensus splice site of the intron. In *LAMA2*, there is a pathogenic variant which also occurred at the fifth nucleotide in intron 37 (c.5562 + 5G>A) (Tezak et al., 2003). The c.5562 + 5G>A resulted in the alternative splicing laminin-α2 with an 11 base-pair insertion in exon 37 (Tezak et al., 2003). We hypothesize that c.4717 + 5G>A may act in a similar way to c.5562 + 5G>A, leading to an aberrant splicing. Beside c.4717 + 5G>A, patient 4 also carried a novel variant c.7156-5_7157delinsT, which is classified as a pathogenic variant with PVS1, PM2, PM3, PM4, PP3, and PP4 supporting (Table 2). This variant creates a truncated protein consisting of 2,386 amino acids (p.R2386*). It means the protein lost 724 amino acids belonging to LG2-LG5 (Figure 5), the region is responsible for linking laminin 211 to the cytoskeleton in muscle cells and is crucial for their interaction with membrane receptors (Nguyen et al., 2019). This study is the first report of c.7156–5_7157delinsT associated with MDC1A patients.

The variant c.4644C>A was reported in *LAMA2* related muscular dystrophy in the study of Töpf et al., however, no clinical and functional information was described (Töpf et al., 2020). Our study is the first detailed clinical report of a MDC1A patient with the variant c.4644C>A. The c.4644C>A creates a premature stop codon (p.C1548*). Patient 5 also carried a deletion of exon 3 which locates in a hotspot area for large deletions (Oliveira et al., 2018). MLPA showed that his mother and brother also carried the deletion of exon 3 (Figure 4). The deletion of exon 3 has been reported in a boy with age of onset at 4-month-old (Oliveira et al., 2014). The *LAMA2* gene sequencing detected a nonsense variant, c.497G>A (p.W166^*^), in the heterozygous and homozygous states in the boy (Oliveira et al., 2014). The authors performed MLPA analysis and long-range PCR to identify the deletion of exon 3 (c.284-4,685_397-146delinsATA (Oliveira et al., 2014). In our study, we could not determine the breakpoints of the exon 3 deletion in patient 5. According to the LOVD database, 21 deletions of exon(s) have been reported in *LAMA2* from 63 reports, and all of them were classified as pathogenic/likely pathogenic variants (Supplementary Table S1). Therefore, in the context of a heterozygous variant being identified in the patients suspected with MDC1A, the clinicians should consider another copy number variation.

After genetic analysis, the patients’ families were provided genetic counseling of potential risks to offspring in the future. During our investigation, the mothers of patient 4 and patient 5 were pregnant and they decided to perform prenatal testing. The results showed that the fetus of family 4 only carried c.4717 + 5G>A in the heterozygous state, therefore, the parents decided to continue the pregnancy. The newborn was given birth with APGAR score of 10 and did not show muscle weakness or symptoms like his brother. The fetus of family 5 carried compound heterozygous variants, including the deletion of exon 3 and c.4644C>A, the parents decided to terminate the pregnancy. Prenatal diagnosis for screening of *LAMA2* mutations has been described since 1998 (Guicheney et al., 1998). Merosin immunohistochemistry of trophoblastic cells was applied for prenatal diagnosis of merosin-deficient muscular dystrophy (Vainzof et al., 2005; Fadiloglu et al., 2018), however, it seems not to be frequently applied in prenatal diagnosis.

Currently, there is no curative treatment for MDC1A. Treatment strategies concentrate on managing symptoms, such as physical therapy, speech therapy, occupational therapy, and diet modifications to alleviate the symptoms (Oliveira et al., 2020; Sousa, 2022). Several drugs have been investigated in different *LAMA2*-CMD mouse models. Omiganil treatment inhibited glyceraldehyde-3-phosphate dehydrogenase-Siah1-mediated apoptosis, protected fibers degradation, improved locomotion in young mice, reduced spine deformation, and prolonged survival in *dy* ^W^ */dy* ^W^ mice (Erb et al., 2009). Doxycycline treatment improved growth, delayed the onset of hindlimb paralysis, reduced the number of inflammatory cells, increased the spontaneous standing movements and Akt phosphorylation, decreased levels of Bax protein, terminal deoxynucleotidyl transferase dUTP nick end labelling-positive myonuclei, and activated caspase-3 in *dy* ^W^/*dy* ^W^ mice (Girgenrath et al., 2009). Losartan treatment enhanced muscle strength, ameliorated fibrosis, inhibited transforming growth factor-β signaling pathway and the mitogen-activated protein kinase cascade in *dy* ^2J^ */dy* ^2J^ mice (Elbaz et al., 2012). Bortezomib treatment increased life span, body weight, locomotion, and muscle morphology, reduced apoptosis and fibrosis, and partially normalized miRNA expression (miR-1 and miR-133a) in *dy* ^3K^/*dy* ^3K^ mice (Körner et al., 2014). Metformin treatment increased weight gain and energy efficiency, enhanced muscle function, and improved skeletal muscle histology in female *dy* ^2J^ */dy* ^2J^ mice (Fontes-Oliveira et al., 2018). N-acetyl-L-cysteine treatment preserved muscle strength, reduced fibrosis, central nucleation, apoptosis, inflammation and oxidative stress, and vitamin E treatment improved skeletal muscle morphological features, reduced inflammation and reactive oxygen species levels in *dy* ^2J^/*dy* ^2J^ mice (Harandi et al., 2020). Vemurafenib treatment improved muscle histopathology and restored the TGF-β/SMAD3 and mTORC1/p70S6K signaling pathways in mouse models, but did not restore the myomatrix (Oliveira-Santos et al., 2023). However, vemurafenib treatment might be combined with other therapies that restore the myomatrix. Several new therapeutic approaches for *LAMA2*-CMD have been investigated, such as integrin-α7β1 regulation, laminin- and cell-based therapies, laminin-111 protein replacement therapy, or laminin-α1 enhancement (Barraza-Flores et al., 2020). The CRISPR/dCas9 system was used to correct a *LAMA2* splice site variant, c.417 + 1G>A, using nonhonmologus end-joining in *dy* ^2J^/*dy* ^2J^ mouse model (Kemaladewi et al., 2017). A restoration of full-length of *LAMA2* was obtained, leading to a functional LAMA2 protein in the mouse model (Kemaladewi et al., 2017). However, due to the large number of *LAMA2* pathogenic variants reported, therapeutic approaches for MDC1A would be mutation-independent and thus favorable to all affected patients. The *LAMA1* is high identical to *LAMA2*, therefore, upregulation of *LAMA1* can be a disease modifier for *LAMA2*-CMD (Kemaladewi et al., 2019). The authors constructed a CRISPR activation system including deactivated Cas9 derived from *Staphylococcus aureus* (*Sa*dCas9), transcriptional activators VP64 (2xVP64) and single-guide RNAs (sgRNAs) targeting the *LAMA1* promoter (Kemaladewi et al., 2019). The CRISPR/dCas9-mediated upregulation of *LAMA1* ameliorated disease symptoms in the *dy* ^2J^ */dy* ^2J^ mouse model (Kemaladewi et al., 2019). Arockiaraj et al. (2023) treated fibroblasts from MDC1A-affected individuals who carried different *LAMA2* pathogenic variants with *Sa*dCas9-2_X_VP64 and three sgRNAs targeting the proximal promoter of *LAMA1*. The results showed a robust upregulation of *LAMA1* expression, and reduction in cell migration and expression of fibroblast growth factor 2 (FGFR2), transforming growth factor-β2 (TGF-β2), and actin alpha 2 (ACTA2). For clinical application in the future, dual adeno-associated virus (AVVs) are needed to package the necessary CRISPRa components (Arockiaraj et al., 2023). However, the high doses of AVV required to achieve clinical efficiency for musculoskeletal disorders resulted in serious adverse events, such as hepatoxiccity and neurotoxicity (Burdett and Nuseibeh, 2023). Therefore, a reduction of the CRISPRa components to decrease the number of AAV is necessary to investigate in the future.

## Conclusion

Targeted sequencing using next-generation sequencing and MLPA are efficient methods for diagnosis CMD as well as MDC1A. By using these methods, we identified abnormalities in the *LAMA2* gene that supported the diagnostic accuracy of MDC1A. The findings significantly contributed in appropriate genetic counseling and prenatal testing for affected families. The effects of the variants identified to the function of LAMA2 need to be evaluated experimentally in the future to improve the understanding of the pathology and develop targeted therapeutic approaches for patients.

## Data Availability

The datasets presented in this study can be found in online repositories. The names of the repository/repositories and accession number(s) can be found in the article/Supplementary Material.
