# Supplementary material for: Merosin-deficient congenital muscular dystrophy type 1a: detection of LAMA2 variants in Vietnamese patients
Source: Front Genet. 2023 Jun 14;14:1183663. doi: 10.3389/fgene.2023.1183663 (PMC10301838; doi:10.3389/fgene.2023.1183663)
Supplement: Supplementary file 1 [file Table2.DOCX]

**Supplementary Table 2. List of primers used in the study**^*^

| **Gene** | **Exon** | **Forward primer sequence (5’-3’)** | **Reverse primer sequence (5’-3’)** | **Length of PCR product (bp)** |
| --- | --- | --- | --- | --- |
| *LAMA2* | 9 | TTGTTTTAGAAATGTTGA | TTACTGGAATAAACAATG | 222 |
| *LAMA2* | 25 | ACCACTTTGGAGACTTTATC | CACCAAACAATGACTAACTT | 263 |
| *LAMA2* | 32 | TCTGCCTGGGATGTTTAG | CTGCCCTGCTTGTGACTG | 226 |
| *LAMA2* | 33 | ATGTTTATGGGATGGAAT | TAGGAAGAAGGTGATTTG | 223 |
| *LAMA2* | 51 | ATGTGGTTGATATTGCTC | AACTTAATCCTTAGCTTT | 228 |
| *LAMA2* | 63 | TGTGTGAACCATCATGAT | GAAATTGTTGCTGGGGTA | 319 |

^*^Primer sequences was obtained from previous research (Guicheney et al., 1998).

Reference:

Guicheney, P., Vignier, N., Zhang, X., He, Y., Cruaud, C., Frey, V., et al. (1998). PCR based mutation screening of the laminin alpha2 chain gene (*LAMA2*): application to prenatal diagnosis and search for founder effects in congenital muscular dystrophy. *J. Med. Genet.* 35, 211–217. doi: 10.1136/jmg.35.3.211.
